# Multifaced roles of PLAC8 in cancer

**DOI:** 10.1186/s40364-021-00329-1

**Published:** 2021-10-09

**Authors:** Misha Mao, Yifan Cheng, Jingjing Yang, Yongxia Chen, Ling Xu, Xun Zhang, Zhaoqing Li, Cong Chen, Siwei Ju, Jichun Zhou, Linbo Wang

**Affiliations:** 1 Department of Surgical Oncology, Sir Run Run Shaw Hospital, Zhejiang University, Zhejiang, 310000 Hangzhou China; 2 Biomedical Research Center and Key Laboratory of Biotherapy of Zhejiang Province, Zhejiang, 310000 Hangzhou China; 3 Department of Gastrointestinal Surgery, Taizhou Hospital of Zhejiang Province Affiliated to Wenzhou Medical University, Taizhou, Zhejiang, 318000 People’s Republic of China

**Keywords:** PLAC8, Tumorigenesis, cancer stemness, Programmed cancer death

## Abstract

The role of *PLAC8* in tumorigenesis has been gradually elucidated with the development of research. Although there are common molecular mechanisms that enforce cell growth, the impact of *PLAC8* is varied and can, in some instances, have opposite effects on tumorigenesis. To systematically understand the role of *PLAC8* in tumors, the molecular functions of *PLAC8* in cancer will be discussed by focusing on how *PLAC8* impacts tumorigenesis when it arises within tumor cells and how these roles can change in different stages of cancer progression with the ultimate goal of suppressing PLAC8-relevant cancer behavior and related pathologies. In addition, we highlight the diversity of *PLAC8* in different tumors and its functional output beyond cancer cell growth. The comprehension of *PLAC8’s* molecular function might provide new target and lead to the development of novel anticancer therapies.

## Introduction

Placenta specific 8 (*PLAC8*), also known as *Onzin, C15, DGIC* and *PNAS-144*, was first identified in genome-wide expression profiling of mid-gestation placentas and embryos using a 15,000 mouse-developmental cDNA microarray [1, 2]. *PLAC8* expression is dynamic during pregnancy and placental development and accumulates in an implantation-dependent manner [1, 3]. *PLAC8* has also been found to be involved in embryo development [4–8]. And *PLAC8* is found to be highly expressed in the endometrium of pregnant *cows* compared to nonpregnant *cows*, and it is upregulated in blastocysts, resulting in *calf* delivery [9–12]. Subsequent research on *PLAC8* was not limited to *animals* but also involved *humans* and many *plants* [13–16]. During the differentiation process of cytotrophoblast cells into interstitial extravillous trophoblast cells, *PLAC8* is greatly induced [17]. To date, *PLAC8* has been determined to be involved in organ development and tumorigenesis [18–21]. In addition, *PLAC8* is a molecular marker to predict prognosis and distinguish between different cell subpopulations [17, 22]. *PLAC8* also plays different roles in a cell- or tissue-type specific manner. Throughout this review, we discuss the structure of PLAC8 and how *PLAC8* evokes widely different responses in tumorigenesis.

## PLAC8 protein

The *PLAC8* gene is located in *human* chromosome 4 and *Mus musculus* chromosome 5, which is one of the placenta-regulatory genes and belongs to the cornifelin family.

**Fig. 1:**
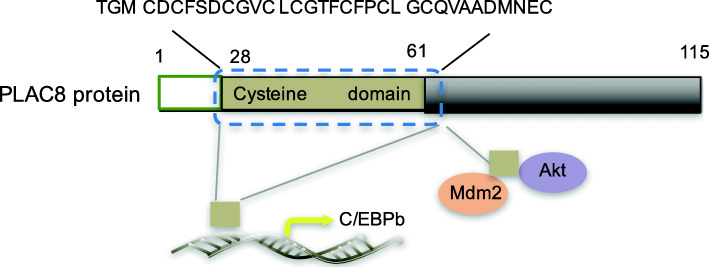
PLAC8 structure. The cysteine-rich domain of the human PLAC8 protein is located between amino acids 28 and 61

The PLAC8 protein contains five exons, coding for a mRNA species of 829 bp and an open reading frame of 115 amino acids [1], which shows a high degree of conservation (83%) between *human*s and *mice* [1, 23]. In addition, *FW2.2-like (FWL)* genes which are identified in *plant* species and *PLAC8* genes, which both contain highly conserved cysteine-rich motifs, share a common ancestor before the divergence between *plants* and *animals* [24]. The first 11 amino acids of this cysteine-rich domain are reported to be required for binding of PLAC8 with Akt1 and MDM-2 protein, and then regulate the activity of Akt1 and MDM-2 [25]. This same region is also found to be required for *PLAC8* transiently binds to the *C/EBPβ* promoter and induce its transcription [26]. In addition, this cysteine-rich domain is called the PLAC8 motif which does not conform to consensus zinc- or RING-finger domains [27, 28]. The PLAC8 motif-containing proteins form a large family and members which can be found in *fungi, algae, higher plants and animals* [29, 30]. In *plants*, *AtPCR1* and *AtPCR2* which contain PLAC8 motif play an important role in transport of heavy metals such as cadmium or zinc [29]. However, our knowledge about the function of PLAC8 motif-containing proteins is very limited. To some extent, although PLAC8 protein has only 115 amino acids (Fig. 1), investigation of its intact domain will help to provide a full understanding of its function and PLAC8 motif-containing proteins.

PLAC8 protein does not have an N-terminal signal peptide, indicating that this protein is not a secretory protein and functions within the cytoplasm or the nucleus [31]. And the precise cellular location of PLAC8 varies greatly depending on its specific context. For instance, the intracellular distribution of the PLAC8 protein is dynamic and regulated in an implantation-dependent manner [32]. PLAC8 is specifically expressed in the interstitial extravillous trophoblast cells on the fetomaternal interface, while its expression is hardly detectable in the endovasculare trophoblast cells [17]. PLAC8 is found exclusively at the apical domain of fully differentiated normal colonic epithelium in both colonocytes and goblet cells [33], and it localizes at the trophoblast cell periphery [17]. In addition, PLAC8 has been found in nasopharyngeal carcinoma and breast cancer cell cytoplasm and membrane [34, 35]. After breast cancer cells acquired drug resistance, PLAC8 accumulated both in nucleus and cytoplasm [36]. In pancreatic cancer cells, PLAC8 is located in the inner plasma membrane [37]. However, in pancreatic ductal adenocarcinoma, PLAC8 is mainly located in lysosomes [38]. The lysosomes contain transporters and participates in the export of molecules [39]. The location of PLAC8 in lysosomes might cause the different location of PLAC8 because of lysosomes interact with other organelles thus leading fusion or non-fusogenic contacts. And these varying localizations may result in its functional differences.

**Fig. 2:**
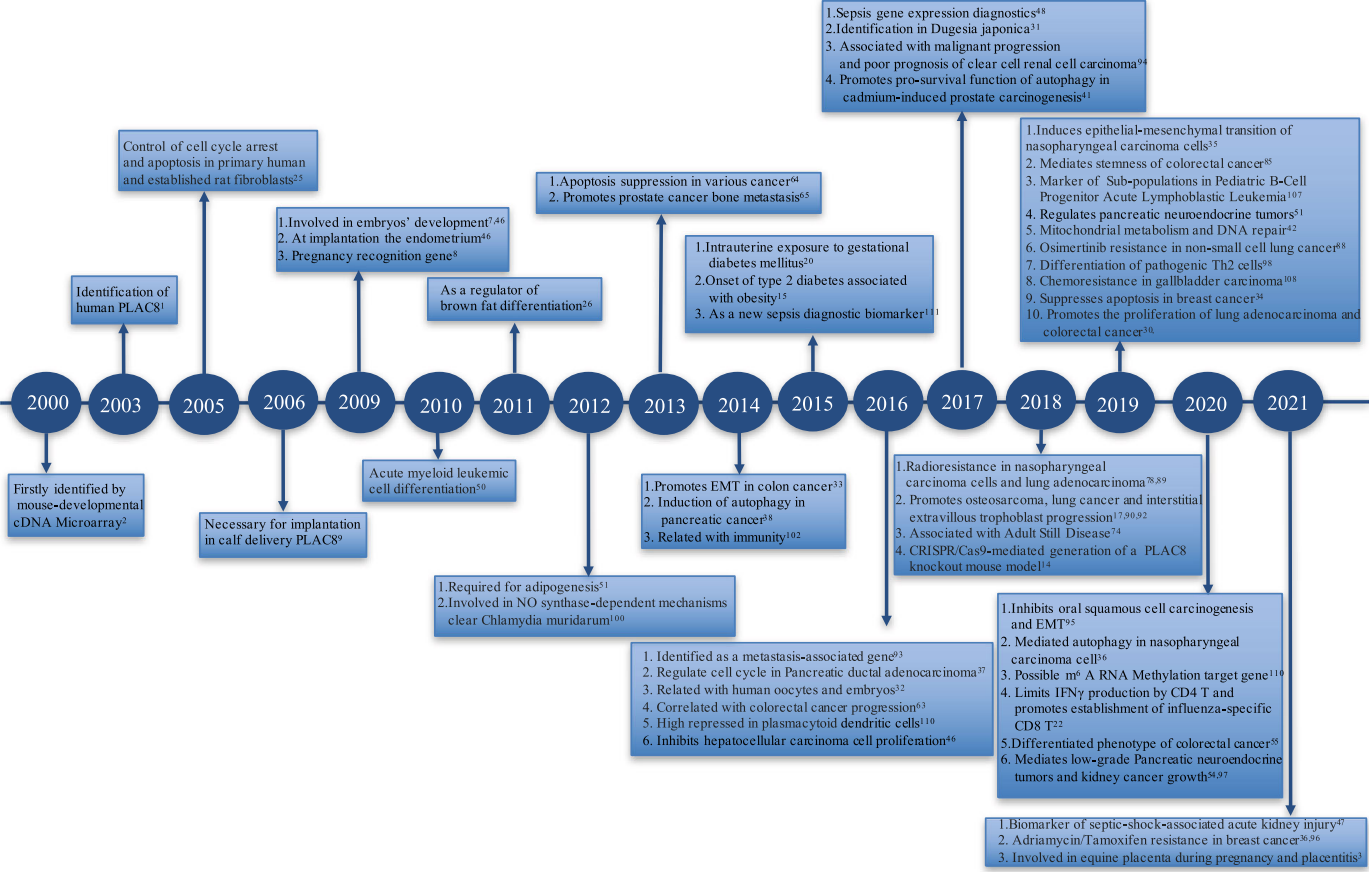
Timeline of *PLAC8* research. A brief history of functional and pharmacological studies of *PLAC8*

Since *PLAC8* was identified 20 years ago, many studies have been performed to identify the characteristics and molecular functions of *PLAC8* in cancer (Fig. 2) [40]. *PLAC8* promotes the growth of tumor cells in prostate cancer cells [41] but significantly inhibits the growth of tumor cells in hepatocellular carcinoma [42]. This interesting phenomenon prompts us to explore the underlying mechanisms and regulatory network of *PLAC8*. Therefore, research on *PLAC8* will help us to further understand the biological characteristics of tumors.

## Connections with cancer

**Fig. 3:**
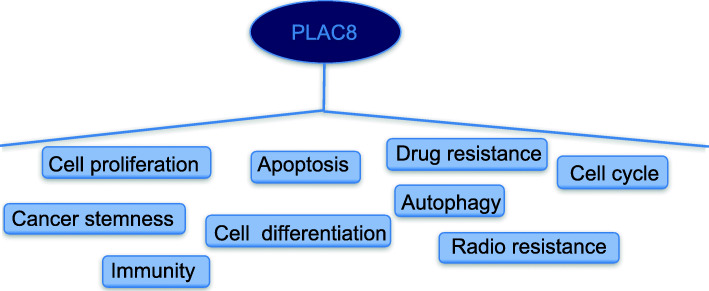
Schematic overview of *PLAC8* functions in cancer progression

As a key regulator of growth in different species, including *fungi* [43]*, plants* [24, 44] *and mammals* [3, 30, 45, 46], *PLAC8* participates in many important physiological activities in different contexts [31, 47–49]. Such as, the ratio of *FAIM3*:*PLAC8* might be a diagnostic biomarker in sepsis [47]. And *PLAC8* is related with septic shock [49]. To date, researchers have also found that *PLAC8* acts as a tumor associated gene that is involved in many cancer processes (Fig. 3) [50–55]. We further discuss the various molecular functions of *PLAC8* in cancer in our review.

### Programed cell death

Programmed cell death, referring to apoptosis, autophagy, programmed necrosis and ferroptosis, may jointly decide the fate of malignant neoplasm cells [56–58]. These forms of programmed cell death balance cell death with cell survival, thus regulating cancer cell fate. Many oncogenes or tumor suppressor genes are linked with tumorigenesis through programmed cell death [59–61]. *PLAC8*, as an oncogene, promotes colorectal and prostate cancer cell growth [62–64]. Cancer growth is always accompanied by programmed cell death. As expected, *PLAC8* regulates cell apoptosis in various cancers [65]. We found that *PLAC8* inhibits breast cancer cell apoptosis, thus promoting cell proliferation [34]. *PLAC8* decreases the sensitivity of lung adenocarcinoma cells to gefitinib-induced apoptosis by reducing the expression of cleaved caspase 3 and cleaved PARP [45]. The mRNA levels of *PLAC8* are increased in stool, and that its increased expression correlates with colorectal cells relapse [63, 66]. PLAC8 is also upregulated in late-stage colorectal patient’s tissues and butyrate which produces microorganisms downregulated PLAC8 expression. And butyrate increased cleaved PARP fragment and then induced apoptosis in colorectal cells [62]. Exception of cancer cells, *PLAC8* can also inhibits cell apoptosis of primary human and established rat fibroblasts via promoting the activation of *MDM-2* and *AKT1* and then inhibiting *p53* [25]. Akt/MDM-2/p53 pathway serves an important role in the regulation of cell apoptosis [67]. And autophagy, is a process that delivers cytoplasmic components to the lysosomes which PLAC8 locates in [38], has opposing and context-dependent roles in cancer [68]. Autophagy induces pancreatic ductal adenocarcinoma cells growth [69]. Pancreatic ductal adenocarcinoma has signature oncogenic mutations of *KRAS* and the inactivation of *p53* [70]. Additionally, in pancreatic ductal adenocarcinoma cell lines, *PLAC8* is cooperatively induced in response to mutations in *KRAS* [71] and *p53* [72] which are the two of the most commonly occurring mutations in cancer. And then *PLAC8* promote pancreatic ductal adenocarcinoma cell lines autophagy thus promoting tumor formation [38]. The oncogenic role of *PLAC8* in inducing the prosurvival function of autophagy protects cells from environmental stress and aids in the transformation of prostate epithelial cells during chronic exposure to cadmium [41]. We previously shown that PLAC8 collaborates with p62 to suppress autophagy in doxorubicin resistant breast cancer cells [36]. PLAC8 inhibits autophagy via the AKT/mTOR pathway in nasopharyngeal carcinoma cells [73]. In addition to cancer, *PLAC8* also enhances autophagy in adult-onset Still’s disease [74] and promotes trophoblast cells autophagy though regulating autophagy-related markers, including LC3B I/II, ATG12 and Beclin-1 [75]. However, the relationship of *PLAC8* with programmed necrosis and ferroptosis, which is a new form of cell death, is still unknown. We previously discussed that an interaction exists between ferroptosis and autophagy [76]. The crosstalk between autophagy and apoptosis regulates testicular injury induced by cadmium via PI3K and a mTOR-independent pathway [77]. Interestingly, *PLAC8* regulates the PI3K pathway and interacts with AKT, which is an important kinase of the PI3K pathway [34, 42, 78]. These results strongly indicate that PLAC8 may be a core regulator in programmed cell death, affect different forms of cell death and decide cancer cell fate. This intriguing contrast in the effects of *PLAC8* on cell fate in different cellular contexts presents attractive possibilities for the development of novel therapies for cancers.

#### Cancer stemness

Stem cells are a population of undifferentiated cells characterized by the ability of self-renewal, such as embryonic stem cells. Studies have shown that the expression of *PLAC8* and several recognized stem cell markers (*NANOG* [79], *SOX2* [80] and *POU5F1* [81]) are commonly highly expressed in embryo development [82]. In *POU5F1*-null embryonic stem cells, *PLAC8* is downregulated [83]. *PLAC8* also may be upstream of *KLF4* which is a stem cell marker [84] in triggering adipogenesis [51]. These studies suggest that *PLAC8* may involve in stem cell progression vis interacting with stem cell markers. Consistant with stem cells, cancer stem cells (CSCs) have the potential to self-renew, and they often appear dormant and resist cancer treatments, such as radiation and chemotherapy, leading to cancer recurrence. Higher PLAC8 expression is found in the sphere-forming colorectal cancer cells than in colorectal cancer cells. And *Id1* gene which can activate the Wnt/β-catenin and Shh signaling pathways promote PLAC8 expression and then maintains cell stemness in colorectal cancer [85]. In non-small cell lung cancer, *PLAC8* promotes the levels of *ALDH1A1* which is a putative marker for CSCs in numerous types of tumors [86–88]. Additionally, *PLAC8* regulates the expression of *POU5F1*, thus increasing stemness during lung adenocarcinoma cell resistance to radiotherapy [89]. And our previous study showed that *KLF4* regulates *PLAC8* transcription in lung cancer cells [90]. These studies strongly indicates that the regulation loop between stem cell markers (*POU5F1* and *KLF4*) and PLAC8 and the various roles of PLAC8 in cancer stemness. The precise association of *PLAC8* with recognized stem cell markers still need further explored. Based on emerging evidence, *PLAC8* may be a promising stemness related marker in tumor initiation and development.

#### Epithelial-mesenchymal transition

Epithelial–mesenchymal transition (EMT) is a cellular process in which cells lose their epithelial characteristics and acquire mesenchymal features that have been associated with metastasis [91]. Studies have shown that *PLAC8* overexpression contributes to MAPK pathway activation and metastatic phenotypes [92] and that *PLAC8* plays a role in the epithelial-mesenchymal transition [93] in different types of cancer. *PLAC8* promotes trophoblast cell, non-small cell lung cancer cell, and clear cell renal cell carcinoma invasion and migration [17, 88, 94, 95]. However, *PLAC8* inhibits oral squamous cell invasion [95]. *PLAC8* reflects the expression of epithelial-mesenchymal related markers including *E-cadherin*, *N-cadherin* and *vimentin* thus involving epithelial-mesenchymal transition process. In breast cancer cells, embryonic kidney 293 T cells, colorectal cancer cells and nasopharyngeal carcinoma cells, *PLAC8* downregulates the level of *E-cadherin* thus regulating cell migration and invasion [34, 35, 96, 97]. On the other hand, *PLAC8* upregulates *N-cadherin* and *vimentin* levels in breast cancer and nasopharyngeal carcinoma cells [34, 73]. Interestingly, *PLAC8* decreases *E-cadherin* expression but increases *P-cadherin* and *vimentin* expression; however, the level of *N-cadherin* is stable in colorectal cancer cells [33]. These studies demonstrate that the molecular function of *PLAC8* varies in different contexts. The difference in cellular position may not be sufficient to explain this phenomenon, and in-depth research is needed in the future. In addition to cadherin family proteins, the abundant expression of *PLAC8* in interstitial extravillous trophoblast cells promotes cell invasion and migration partially by upregulating the activation of *RAC1* and *CDC42* without change their expression [17]. *PLAC8* not only promotes EMT progression but is also involved in cancer metastasis, such as bone metastasis in prostate cancer cells and lung metastasis in colorectal cancer cells *in vivo* [62, 64]. Taken together, *PLAC8* may reflect epithelial-mesenchymal related genes thus involving EMT progression and cancer metastasis. Additionally, the expression of *PLAC8* can predict of changes in EMT markers, including *E-cadherin*, *N-cadherin* and *vimentin* and be the hallmark of EMT progression.

#### Cancer immunity

*PLAC8* exists in a variety of immune cells and the level of *PLAC8* varies in different immune cells. *PLAC8* is higher expressed by Th1 CD4 T-cells compared to Th2, Th17 and iTreg CD4 T-cells [22]. In addition, *PLAC8* is relatively highly expressed in airway T helper 2 (Th2) cells which play a pathogenic role in allergies [98]. *PLAC8* is robustly downregulated in CD39^+^ human regulatory T-cells [99]. In addition to being expressed in immune cells, *PLAC8* also interacts with immune factors and regulates inflammation. For example, *PLAC8* suppresses the production of the pro-inflammatory cytokines, IL-1b and IL-18, via enhancement of autophagy in adult-onset Still’s disease [74]. PLAC8 is important for suppressing IFNγ production by IL-12 stimulation in CD4 T-cell [22]. And CD4 T-cell expression of PLAC8 correlates with potent termination of Chlamydia replication and relative independence from IFNγ pretreatment of epithelial monolayers [100, 101]. And Chlamydia-specific CD8 T-cell clones do not express PLAC8 [102], but PLAC8 also promotes effector CD8 T-cell establishment through a T cell-intrinsic mechanism. In addition, *PLAC8* is identified in placental functions, and *PLAC8* is relatively higher in placentitis cells [103]. *PLAC8* mRNA is also increased in the myometrium of adenomyosis patients, indicating the role of the immune response in the myometrium of women with adenomyosis [104]. These evidences suggest that *PLAC8* may play an important role in immune system [31, 105, 106]. Determining factors that regulate *PLAC8* expression in T cells may help to identify how it can be utilized therapeutically during T cell-driven inflammation, and the functions of *PLAC8* in the immune system, especially in the regulation of different populations of immune cells, need to be explored further.

When referred to cancer immunity, *PLAC8* is found to be most intensively expressed in the FXIII-A dim subgroup and helps to define three novel subpopulations in pediatric B-cell progenitor acute lymphoblastic leukemia [107]. And RNA sequencing data of clear cell renal cell carcinoma has shown that *PLAC8* is mainly involved in immunity-related pathways [94]. With unbiased RNA sequence analysis, *CXCL5*, which is an inflammatory mediator, has been identified as one of the downstream targets of *PLAC8* overexpression in osteosarcoma [92]. Gong et al. found that *PLAC8* is abnormally overexpressed in gallbladder carcinoma cells and that its expression positively correlates with *PD-L1* expression, which is the main checkpoint of the immune system [108]. However, time and more research will begin to address questions that how *PLAC8* involves cancer immunity. While these findings were initially unexpected, *PLAC8* is an immune-related gene and may be a targeting gene for immune reactions in cancer.

#### Drug resistance

In the *ericoid mycorrhizal fungus, Oidiodendron maius*, PLAC8-containing proteins have been reported to be involved in cadmium tolerance [28]. Additionally, specifically targeting *PLAC8* may affect prostate carcinogenesis in humans, and *PLAC8* activation may be used as a biomarker for the early detection of prostate cancer in cadmium-exposed populations [41]. These findings indicate that the expression of *PLAC8* might be altered upon exposure to certain drugs. Drug resistance is one of the main reasons for the failure of tumor therapy, which greatly limits the choice and use of cancer drugs. Researchers have demonstrated that *PLAC8* is related to multidrug resistance in various cancers. In nasopharyngeal carcinoma cells, knockout of *PLAC8* radiosensitizes nasopharyngeal carcinoma cells by activating the PI3K/AKT/GSK3β pathway [78]. Our study found that overexpression of *PLAC8* can promote tamoxifen resistance in breast cancer and that the expression of *PLAC8* can be reduced by curcumin [96]. In addition to endocrine resistance, *PLAC8* regulates *RAC1* levels, and another study has reported that *RAC1* promotes breast cancer chemoresistance by influencing DNA damage repair [17, 109]. These findings indicate that *PLAC8* may predict multidrug resistance in breast cancer. In non-small cell lung cancer, overexpression of *PLAC8* in parental cells markedly decreases osimertinib sensitivity [88]. Enhanced sensitivity to cisplatin treatment following silencing of *PLAC8* in clear cell renal cell carcinoma cells suggests a potential therapeutic target of *PLAC8* [94]. *PLAC8* overexpression decreases sensitivity to gemcitabine and oxaliplatin in gallbladder carcinoma cells [108]. Overexpression of *PLAC8* significantly decreases the sensitivity of lung adenocarcinoma to gefitinib [45]. Taken together, these results suggest that *PLAC8* may predict drug resistance in various cancer cells and be a promising therapeutic target.

#### Other diseases

In addition to its important role in tumors, PLAC8 also participates in other disease processes, such as respiratory diseases and some infectious diseases [98, 102, 110]. For example, PLAC8 is upregulated in activated monocytes and in monocytes isolated from active ASD patients [74]. In addition, many studies have shown that PLAC8 is related to glucose metabolism [26]. However, animal models have shown that PLAC8 is expressed at different levels in F344-fa and F344-fa-nidd2 rats and is closely related to obesity and glucose loading [15]. The AIM3:PLAC8 ratio is a candidate biomarker that can be used to assist in the rapid diagnosis of CAP on ICU admission [111]. The study of PLAC8 in different systemic diseases in humans may help to further understand the function of this gene.

## Overview of the PLAC8-regulated network

**Fig. 4:**
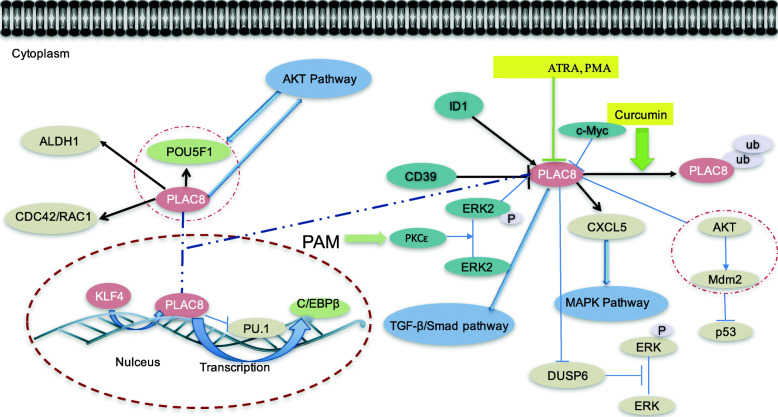
Signaling pathways and genes controlling *PLAC8* expression and its regulatory system. *PLAC8* regulation is driven by different factors in both the nucleus and cytoplasm. It is important to point out that published mechanisms of *PLAC8* regulation are not yet completely understood. Studies have shown that growth-related signaling pathways, such as the AKT, MAPK and TGF-β/Smad pathways, interact with *PLAC8*. Some drugs, such as curcumin and PAM, directly and indirectly affect PLAC8 levels. In addition, *PLAC8*, as a transcription factor, promotes *C/EBPβ* transcription and inhibits *PU.1* transcription. The dashed lines depict mechanisms that are not completely understood. *C/EBPβ*, enhancer-binding protein β; *ALDH1A1*, aldehyde dehydrogenase 1 family member A1; *CDC42*, cell division control protein 42; *POU5F1*, POU Class 5 homeobox 1; *RAC1,* ras-related C3 botulinum toxin substrate 1; *KLF4,* Kruppel-like factor-4; *PLAC8*, placenta-specific gene 8; *PU.1*, Spi-1 proto-oncogene; *CD98*, ectonucleoside triphosphate diphosphohydrolase 1; *ID1*, inhibitor of differentiationId-1; *PKCɛ*, protein kinase C ɛ; *ERK2*, extracellular regulated protein kinases 2; *c-Myc*, cellular myelocytomatosis viral oncogene; *CXCL5*, C-X-C motif chemokine 5; *DUSP6*, dual specificity phosphatase 6; *MDM-2*, murine double minute 2; *p53*, tumor protein 53

There is mounting evidence of the potential role of PLAC8-regulated network in cancer (Fig. 4) [104, 111, 112]. *PLAC8* can be regulated at the transcriptional level. For example, *PLAC8* is involved in pro-mesonephros regulation, and *PAX2* regulates the transcription of *PLAC8* [113]. *PLAC8* is upregulated by IFNT [114], and the expression of *PLAC8* is upregulated under hypoxia [17]. *PLAC8* acts as a transcription factor involved in the expression of different genes. In CD4 T cells, *PLAC8* suppresses IL-12-induced IFNγ production at the transcriptional level [22]. *PLAC8* binds to the *C/EBPβ* promoter to induce its transcription [26]. *PLAC8* activates the Akt/MDM-2 pathway, ultimately leading to an inability to upregulate *p53*. In addition, PLAC8 directly interacts with MDM-2 and Akt, thereby influencing the localization of both proteins [25]. In functional extravillous trophoblasts, PLAC8 colocalizes with p53 and regulates p53 expression at the posttranslational level [75]. In addition, the expression of *PLAC8* can be reduced by curcumin in tamoxifen resistant breast cancer [96]. And butyrate reduced the expression of *PLAC8* in colorectal cancer cells [62]. In acute myeloid leukemic cell lines, all-trans retinoic acid (ATRA) and phorbol 12-myristate 13-acetate (PMA) downregulate *PLAC8* expression though PKCɛ-ERK2 signaling pathway [50]. As shown in Fig. 3, *PLAC8* interacts with tumor-related genes both at the transcriptional and posttranscriptional levels, thereby playing a functional role in cancer progression.

## Conclusion and perspectives

**Fig. 5:**
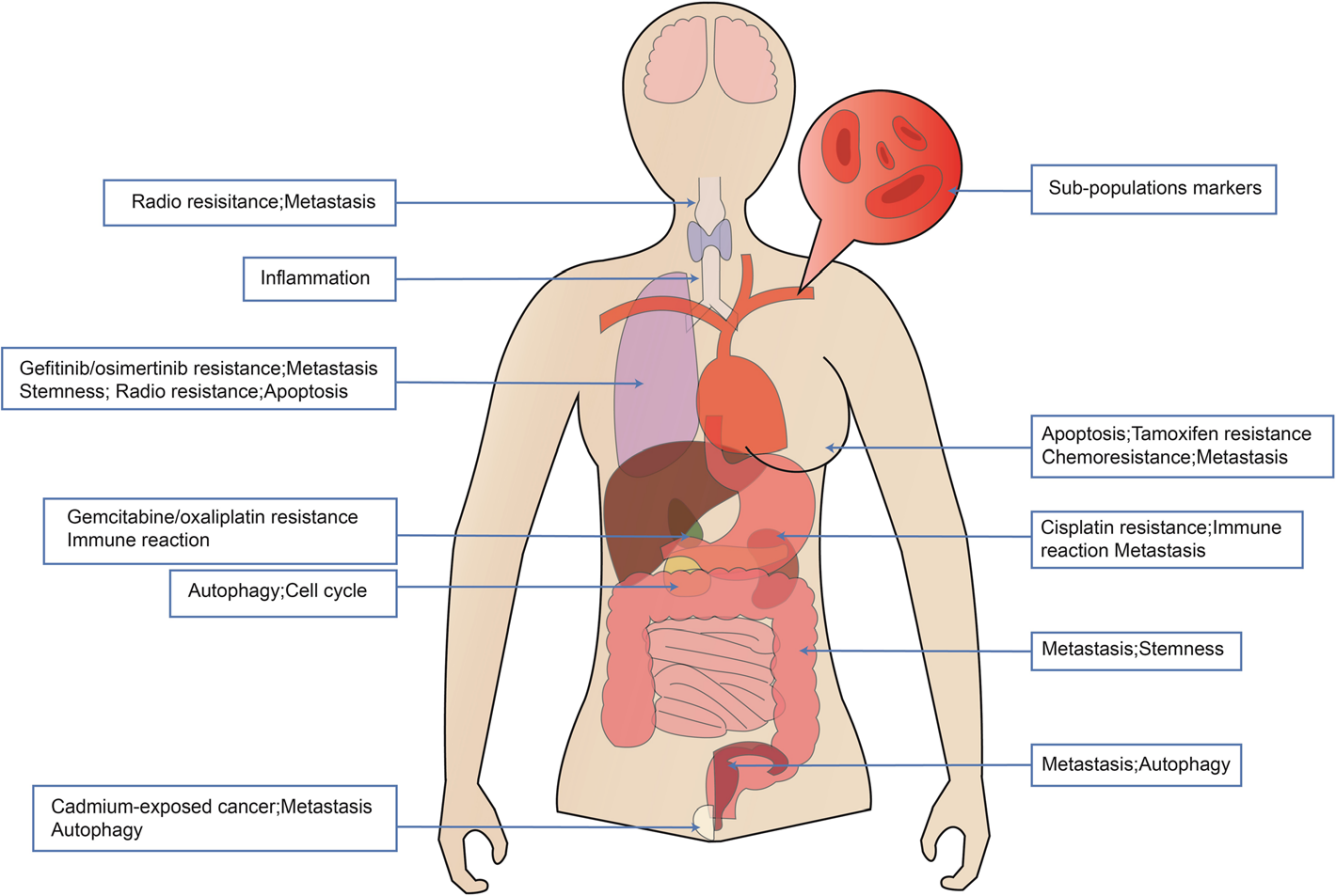
Epidemiological data and functional evidence of *PLAC8* in tumor types

Our understanding of the molecular mechanisms of *PLAC8* has expanded over the last decade, and this knowledge has been used to build better models that allow us to unravel the complicated role of the *PLAC8* gene in human diseases. Furthermore, these studies have led to the identification of putative therapeutics to target *PLAC8*. While *PLAC8* accumulates in most tumor cells, it tends to contribute to tumor progression by inducing tumorigenesis, immune reactions, chemoresistance and metastasis. As discussed above, *PLAC8* has been identified in breast cancer, prostate cancer, lung cancer gallbladder cancer and nasopharyngeal cancer (Fig. 5). The molecular functions of *PLAC8* in the brain, gastric carcinoma and osteocarcinoma remain unknown and need to be explored. Based on these studies, we suggest that *PLAC8* may be a promising marker and predictor for clinical drug selection, immunotherapy response and tumor prognosis. The precise roles of *PLAC8* in different cancers vary, and its underlying mechanisms should be determined in the future. In addition, the relative network related to *PLAC8* is still not clear. Therefore, the mechanisms by which *PLAC8* selects its downstream partners and is reflected by other genes may reveal new players and mechanisms by which *PLAC8* orchestrates cancer cell behavior, thereby suggesting new targets for therapy. Another aspect that deserves attention is to understand the functional structure of each region of the PLAC8 protein, which will help to comprehend the related molecular mechanism of the protein. Further characterization of the PLAC8 protein in different cell types is paramount not only to enrich our understanding of this gene in normal physiology but also to enhance our ability to target it to reduce cancer progression. Thus, the precise roles of *PLAC8* in different forms of programmed cancer death need to be discovered in the future.

## Data Availability

Not applicable.
